# Notch signaling enhances PASMC proliferation and vascular remodeling in CTEPH

**DOI:** 10.1515/biol-2025-1251

**Published:** 2026-02-25

**Authors:** Salamaiti Aimaier, Ailiman Mahemuti, Refukaiti Abuduhalike, Wen-kui Lu, Yu-jun Guo, Li Zhao

**Affiliations:** Department of Heart Failure, First Affiliated Hospital of Xinjiang Medical University, 830054, Urumqi, China

**Keywords:** chronic thromboembolic pulmonary hypertension, Notch signaling, pulmonary artery smooth muscle cells, proliferation, vascular remodeling

## Abstract

The Notch signaling pathway is implicated in pulmonary vascular remodeling (PVR) in Chronic Thromboembolic Pulmonary Hypertension (CTEPH). This study investigated the role of Notch signaling in PVR using a rat model of CTEPH and performed measurements including Right Ventricular Systolic Pressure (RVSP), Mean Pulmonary Arterial Pressure (MPAP), and histological analysis of pulmonary small arteries. Western blot analysis revealed elevated levels of Notch1, Notch3, Jagged1, and Hes1 proteins in CTEPH rats. Furthermore, the Notch pathway proteins (Notch1, Notch3, Jagged1, and Hes1) were even more elevated in the CTEPH + VPA group. In contrast, protein levels were reduced in the CTEPH + DAPT (a *γ*-secretase inhibitor) group. The medial wall thickness percentage (MWT%) and markers of smooth muscle cell proliferation, including PCNA and *α*-SMA, were significantly increased in the CTEPH and CTEPH + VPA groups and decreased after DAPT treatment. These findings suggest that activation of the Notch signaling pathway promotes the proliferation of Pulmonary Artery Smooth Muscle Cells (PASMCs), contributing to PVR in CTEPH. Inhibition of Notch signaling with DAPT mitigated vascular remodeling, suggesting a potential therapeutic target for CTEPH.

## 1 Introduction

Chronic thromboembolic pulmonary hypertension (CTEPH), a severe and progressive form of pulmonary hypertension (PH), results from the obstruction of pulmonary arteries due to unresolved pulmonary thromboembolism (PTE) [1]. CTEPH develops from the persistence of organized blood clots, causing vascular obstruction and subsequent remodeling of the pulmonary vasculature [2], 3]. These changes lead to elevated pulmonary vascular resistance (PVR) and pulmonary artery pressure (PAP), and in severe cases, can cause ventricular failure and even death. It was reported that the 2-year cumulative incidence of CTEPH following symptomatic acute PTE ranges from 0.1 % to 11.8 % [4]. Previous meta-analyses have shown that the incidence of CTEPH in all PTE patients is 0.56 %, and it is 3.2 % among PTE survivors at 6 months [5]. Another large-scale prospective observational investigation showed that the 2-year cumulative incidence of CTEPH in acute PTE patients is 2.3 % [6]. Consequently, the annual economic burden of CTEPH is substantial, encompassing costs related to prolonged hospitalizations, complex treatments, and lost productivity, amounting to billions of dollars globally [7], 8], thus urging the exploration and elucidation of the pathogenesis and therapeutic strategies of CTEPH.

The pathogenesis of CTEPH is critically dependent on the proliferation and dysfunction of Pulmonary Artery Smooth Muscle Cells (PASMCs) [9]. In patients with CTEPH, PASMCs exhibit abnormal proliferative and migratory capabilities, which are closely associated with pulmonary vascular remodeling [10], 11]. The phenotypic switch of these cells from a contractile to a synthetic phenotype leads to excessive deposition of extracellular matrix and thickening of the vascular wall, thereby increasing pulmonary arterial resistance [12]. Specifically, PASMCs contribute to the thickening of the vascular walls, increasing resistance to blood flow, and exacerbating the condition [12], 13]. PASMCs contribute to inflammation and vascular remodeling by secreting cytokines and growth factors, such as transforming growth factor-*β* (TGF-*β*) [10] and platelet-derived growth factor (PDGF) [14]. These processes not only exacerbate pulmonary artery stenosis but may also promote thrombus formation and progression. Diverse signaling pathways, such as PDGF, TGF-*β*, and Notch pathways, are involved in the health maintenance of PASMCs [15], and the Notch signaling pathway is one of the critical players in vascular remodeling associated with CTEPH [16].

The Notch signaling pathway, a highly conserved cell-to-cell communication mechanism that controls cell differentiation, proliferation, and apoptosis by regulating direct cell-to-cell contact, plays a crucial role in embryonic development, tissue homeostasis, and disease pathogenesis [17]. The main components of the Notch signaling pathway include the Notch receptors (Notch1–4) and their ligands (such as members of the Delta-like and Jagged families) [18]. Proteolytic events are triggered when a ligand binds to a receptor, releasing the Notch intracellular domain (NICD). Subsequently, NICD translocates to the nucleus, binds to transcription factors, and regulates the expression of downstream target genes [19]. Under normal physiological conditions, the Notch signaling pathway regulates the interactions between cells to maintain the health of cells [20], 21]. However, in the pathological state of CTEPH, abnormal activation or inhibition of the Notch signaling pathway may lead to an imbalance in vascular remodeling. Specifically, dysregulation of the Notch signaling pathway may affect the proliferation, migration, and phenotypic switching of PASMCs [22], 23]. It involves the interaction between Notch receptors (such as Notch1 and Notch3) and their ligands (such as Jagged1), leading to the activation of downstream transcription factors, including Hes1 [24]. In the context of CTEPH, Notch signaling is believed to influence PASMC proliferation and contribute to the remodeling of pulmonary vasculature [25]. However, the precise mechanisms through which Notch signaling mediates these effects remain incompletely understood.

To address this gap, we utilized a rat model of CTEPH to investigate Notch signaling’s role in PASMC proliferation and vascular remodeling. We assessed the hemodynamic changes by measuring right ventricular systolic pressure (RVSP) and mean pulmonary arterial pressure (MPAP) in the rat model. Additionally, histological and immunohistochemical analyses were conducted to observe the pathological alterations in the pulmonary small arteries. Western blotting was employed to measure the protein levels of Notch1, Notch3, Jagged1, and Hes1 in the pulmonary arteries of the rats. Our results indicate a significant elevation in the expression levels of Notch1, Notch3, Jagged1, and Hes1 in the CTEPH model. Enhanced Notch/Jagged/Hes signaling activity appears to promote PASMC proliferation and contribute to the pulmonary vascular remodeling characteristic of CTEPH. Furthermore, DAPT, a *γ*-secretase inhibitor known to block Notch signaling, attenuated these effects and reduced vascular remodeling. These findings advance our understanding of the role of Notch signaling in CTEPH and highlight its potential as a therapeutic target for mitigating disease progression.

## 2 Materials and methods

### 2.1 Overall experimental design

**Figure 1:**
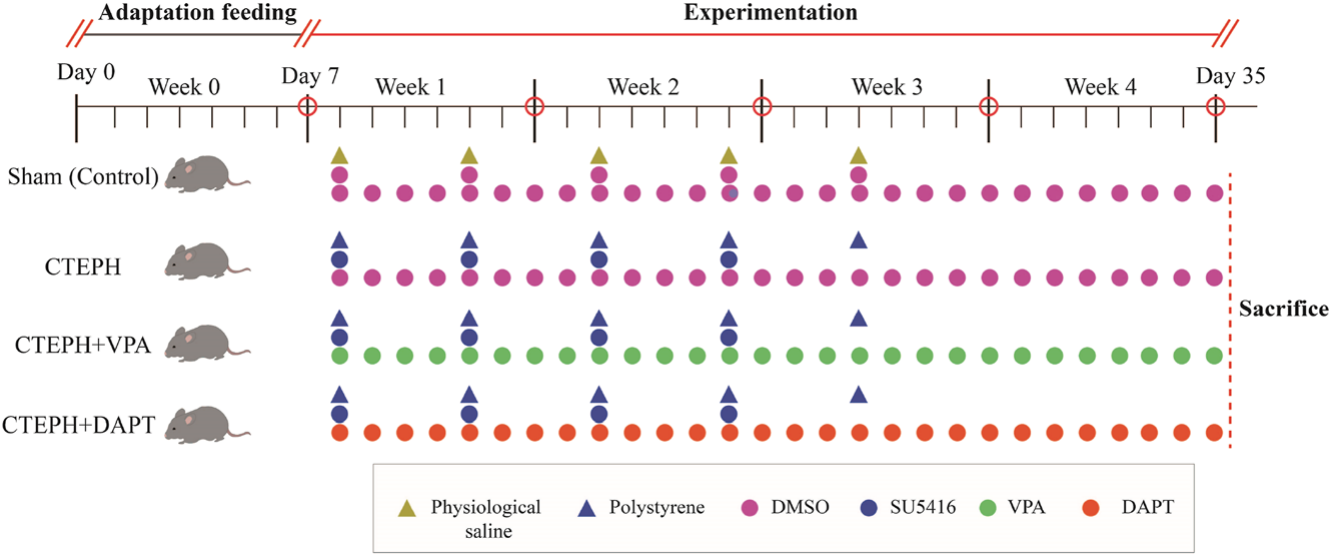
Experimental workflow for the CTEPH rat model and treatment groups. (1) Sham (control) group (*n* = 8): physiological saline (2 mL/kg) was injected into the tail vein every 4 days for a total of five injections. The same volume of DMSO solvent (2 mL/kg) was injected subcutaneously. Starting from the first week of the experiment, the same volume of DMSO solvent (2 mL/kg) was injected intraperitoneally every day; (2) CTEPH group (*n* = 8) was administered 1 × 10^6^ microspheres/kg via tail vein injection every 4 days for a total of five injections. At the same time, they received subcutaneous injections of 20 mg/kg SU5416 every 4 days for a total of four injections. Starting from the first week of the experiment, the same volume of DMSO solvent was injected intraperitoneally every day; (3) the PH + VPA group (*n* = 8) was administered 1 × 10^6^ microspheres/kg via tail vein injection every 4 days for a total of five injections. At the same time, they received subcutaneous injections of 20 mg/kg SU5416 every 4 days for a total of four injections. Starting from the first week of the experiment, VPA (300 mg/kg/day) was injected intraperitoneally every day for 4 weeks; (4) the PH + DAPT group (*n* = 8) was administered 1 × 10^6^ microspheres/kg via tail vein injection every 4 days for a total of five injections. They received subcutaneous injections of 20 mg/kg SU5416 every 4 days for four injections. Starting from the first week of model induction, DAPT (10 mg/kg/day) was administered intraperitoneally every day for 4 weeks. The animals of all the groups were sacrificed after the fourth week. The corresponding methods sections presented the detailed procedures and methods for preparing and applying related solutions.

The main procedure of the experiment workflow is presented in Figure 1. We purchased 32 healthy SPF-grade Sprague-Dawley (SD) male rats from the Zhejiang Center of Laboratory Animals for this study. These rats were chosen for their age (4–6 weeks) and weight (250–300 g). The rats were housed at a temperature of 23 ± 3 °C with 40–70 % humidity, and we followed a previous report for the animal grouping [26]. The rats were maintained on a 12-h light/dark cycle with *ad libitum* access to food and water and were allowed to acclimate for one week before the start of the study.

**Ethical approval:** The research related to animal use has been complied with all the relevant national regulations and institutional policies for the care and use of animals, and has been approved by the Institutional Animal Care and Use Committee, Zhejiang Center of Laboratory Animals, Hangzhou, China (approval number ZJCLA-IACUC-20020180).

### 2.2 Reagent preparation

Firstly, 360 mg of SU5416 (a vascular endothelial growth factor (VEGF) receptor inhibitor) in 20 mL of DMSO to obtain a concentration of 18 mg/mL, and the solution was stored in the dark. Before injection, the solution was diluted with physiological saline to a final concentration of 4.5 mg/mL. To prepare 10 % neutral formalin, we diluted the formalin solution with 1× PBS and stored it at 4 °C. To prepare the sodium valproate solution (valproic acid sodium, VPA), 4.2 g of VPA was dissolved in 33.61 mL of 1× PBS to achieve a 125 mg/mL concentration, and the solution was stored at 4 °C. To prepare the *γ*-secretase inhibitor (DAPT), 140 mg of DAPT was dissolved in 7 mL of DMSO to obtain a concentration of 20 mg/mL, and we diluted it with sterile physiological saline to a final concentration of 5 mg/mL before injection.

### 2.3 Animal grouping, model preparation and experimentation

The SD rats were randomly assigned to groups using a random number table, and rats in each group were treated as follows:

(1) **Sham Group (*n* = 8):** Rats received tail vein injections of 2 mL/kg physiological saline once every 4 days for five injections, along with subcutaneous injections of 2 mL/kg of solvent DMSO. From the first week of the experiment, rats were administered daily intraperitoneal injections of 2 mL/kg of the solvent DMSO. Animals were sacrificed after 4 weeks.
(2) **CTEPH Group (*n* = 8):** Rats were injected with a suspension of 1 × 10^6^ 50 µm polystyrene microspheres/kg via the tail vein once every 4 days for a total of five injections, along with subcutaneous injections of 20 mg/kg SU5416 (dissolved in 20 mg/mL DMSO), once every 4 days for a total of four injections. From the first week of the experiment, rats were administered daily intraperitoneal injections of the same volume of solvent DMSO. Animals were sacrificed after four weeks.
(3) **CTEPH + VPA Group (*n* = 8):** Rats were treated as in the CTEPH Model Group with injections of 1 × 10^6^ 50 µm polystyrene microspheres/kg via the tail vein and subcutaneous injections of 20 mg/kg SU5416 (dissolved in 20 mg/mL DMSO) following a previous report [27]. From the first week of the experiment, rats received daily intraperitoneal injections of VPA (300 mg/kg/day, dissolved in 20 mg/mL DMSO) for 4 weeks. Animals were sacrificed after four weeks.
(4) **CTEPH + DAPT Group (*n* = 8):** Rats were treated as in the CTEPH Model Group with injections of 1 × 10^6^ 50 µm polystyrene microspheres/kg via the tail vein and subcutaneous injections of 20 mg/kg SU5416 (dissolved in 20 mg/mL DMSO) following a previous report [27]. From the first week of modeling, rats received daily intraperitoneal injections of 10 mg/kg/day DAPT (dissolved in 20 mg/mL DMSO) for 4 weeks according to prior studies [28], 29]. Animals were sacrificed after 4 weeks.

### 2.4 Measurement of right ventricular systolic pressure (RVSP) and mean pulmonary artery pressure (MPAP)

At the end of week 4 of the experiment, the animals were anesthetized with 2 % sodium pentobarbital administered via intraperitoneal injection. The animals were then placed in a supine position, and the right external jugular vein was carefully isolated. A PE-50 polyvinyl chloride catheter, connected to a pressure transducer, was inserted through the right external jugular vein and advanced gradually. The position of the catheter was confirmed by monitoring changes in the pressure waveform. Once the catheter reached the pulmonary artery via the right ventricle, pulmonary arterial pressure (PAP) was recorded. Subsequently, right ventricular systolic pressure (RVSP) was measured for each group of rats.

### 2.5 HE staining and pathological observation

The HE staining and observation were conducted according to a previously reported method [30]. Lung tissues, including small pulmonary arteries, from each group of rats, were fixed in formalin and processed through routine dehydration, paraffin embedding, sectioning, and hematoxylin and eosin (HE) staining. After natural air drying, pathological changes in the pulmonary vasculature were examined and photographed at 100×, 200×, and 400× magnifications using a light microscope (CKX41-A22PHP, Olympus, Japan). Tissue sections were approximately 4 µm in thickness. Ten vessels with diameters ranging from 50 to 150 µm were randomly selected for measurement. The medial wall thickness (MWT) and external diameter (ED) of the small pulmonary arteries were determined, and the percentage of MWT relative to ED (MWT%) was calculated using the formula: MWT% = (2 × MWT/ED) × 100 %.

### 2.6 Immunohistochemistry and observation

Immunohistochemical experimentation was conducted according to a previous report [31], and applied anti-PCNA (1:200, cat. no. 13110; Cell Signaling Technology, Inc.) and HRP-conjugated secondary antibody (Sino Biological, 1: 2,000). Lung tissues, including small pulmonary arteries, from each group were fixed in formalin and processed through routine dehydration, clearing, paraffin embedding, and sectioning. After incubation with primary and secondary antibodies, the tissue sections were incubated at 37 °C overnight. The expression and localization of SM*α*-actin and PCNA were then examined under an optical microscope (Leica DMIRB, Germany). Protein expression in small pulmonary arteries was evaluated using a manual scoring system based on staining intensity and the percentage of positive cells. Staining intensity was graded as follows: negative = 0, light yellow = 1, light brown = 2, dark brown = 3. The extent of positive staining was scored as follows: 76–100 % = 4, 51–75 % = 3, 26–50 % = 2, 1–25 % = 1, 0 % = 0.

### 2.7 Western blot analysis of Notch1, Notch3, Jagged1, and Hes1 proteins in small pulmonary arteries

Pulmonary arteriolar tissues from each group of rats were collected, and total proteins were extracted. Protein concentration was determined using the BCA (bicinchoninic acid) protein assay kit (Beyotime, China) on a spectrophotometer (Tianmei Company, Beijing, China). Specifically, 100 mg of lung tissue was homogenized and mixed with 1 mL of cell lysis buffer (PMSF: RIPA = 1:100), incubated on ice for 1 h, sonicated, and centrifuged. The supernatant was transferred to a new 1.5 mL EP tube. Protein concentration in the supernatant was measured using a BCA protein assay kit, with absorbance quantified using a spectrophotometer. Protein samples were mixed with loading buffer, boiled at 100 °C for 10 min, and stored on ice. For electrophoresis, 80 µg of protein per sample was loaded onto the gel, and proteins were subsequently transferred to PVDF membranes. The membranes were blocked with 10 % non-fat milk or 5 % BSA at room temperature for 2 h. In the Western blot experiment, the primary antibodies used and their respective dilution ratios were as follows: *β*-actin was diluted at a ratio of 1:1,000, Notch1 at 1:500, Notch3 at 1:500, Jagged1 at 1:500, and Hes1 at 1:400. The secondary antibodies employed were Goat Anti-Mouse IgG H&L (HRP) for *β*-actin, which was diluted at a ratio of 1:10,000, and Goat Anti-Rabbit IgG H&L (HRP) for the other primary antibodies (Notch1, Notch3, Jagged1, and Hes1, all of which were diluted at a ratio of 1: 5,000) were applied and incubated at room temperature for 1 h. The membranes were washed again with TBST (5 min × 3 times). A chemiluminescent detection solution was prepared and applied to the membranes, and the results were captured by exposure and imaging.

### 2.8 Statistical analysis

Statistical analysis was performed using SPSS 19.0 (SPSS Inc., Chicago, IL, USA), and graphical representations were created with GraphPad Prism 5.0 (GraphPad Software Inc.; San Diego, CA, USA). In the inter-group difference analysis, the normality of the data for each group was first assessed using the Shapiro-Wilk test. If the data followed a normal distribution, pairwise ANOVA was performed to evaluate the significance of inter-group differences. If the data did not follow a normal distribution, the Kruskal-Wallis test was applied. A *p*-value greater than 0.05 was considered not significant, while a *p*-value less than 0.01 was deemed significant and marked with “**”. For p-values between 0.01 and 0.05, significance was indicated with “*” and these values were annotated in the figures. Normally distributed continuous data, presented as mean ± standard deviation (SD), were analyzed using one-way ANOVA followed by Tukey’s post-hoc tests (LSD for equal variances or Dunnett’s for unequal variances). For non-parametric comparisons among multiple groups, the Kruskal-Wallis test was used, followed by pairwise comparisons within the same framework. Specifically, hemodynamic data (right ventricular systolic pressure and mean pulmonary artery pressure) were evaluated via one-way ANOVA followed by Tukey’s post-hoc test, after confirming normality and homogeneity of variances using the Shapiro-Wilk and Levene’s tests. Immunohistochemistry data were analyzed using the Kruskal-Wallis test. Western blot analysis of Notch1, Notch3, Jagged1, and Hes1 proteins utilized two-way ANOVA to account for group and replicate effects. A significance level of *α* = 0.05 was adopted throughout, with data reported as mean ± SD for normally distributed variables.

## 3 Results

### 3.1 General health conditions of rats in each group

In the Sham group, the rats displayed good overall health, characterized by smooth and shiny fur, gradual weight gain, and no mortality. In contrast, the CTEPH model group exhibited poor general condition, with rough, dull fur, progressive weight loss, reduced activity, and the death of three rats.

**Figure 2:**
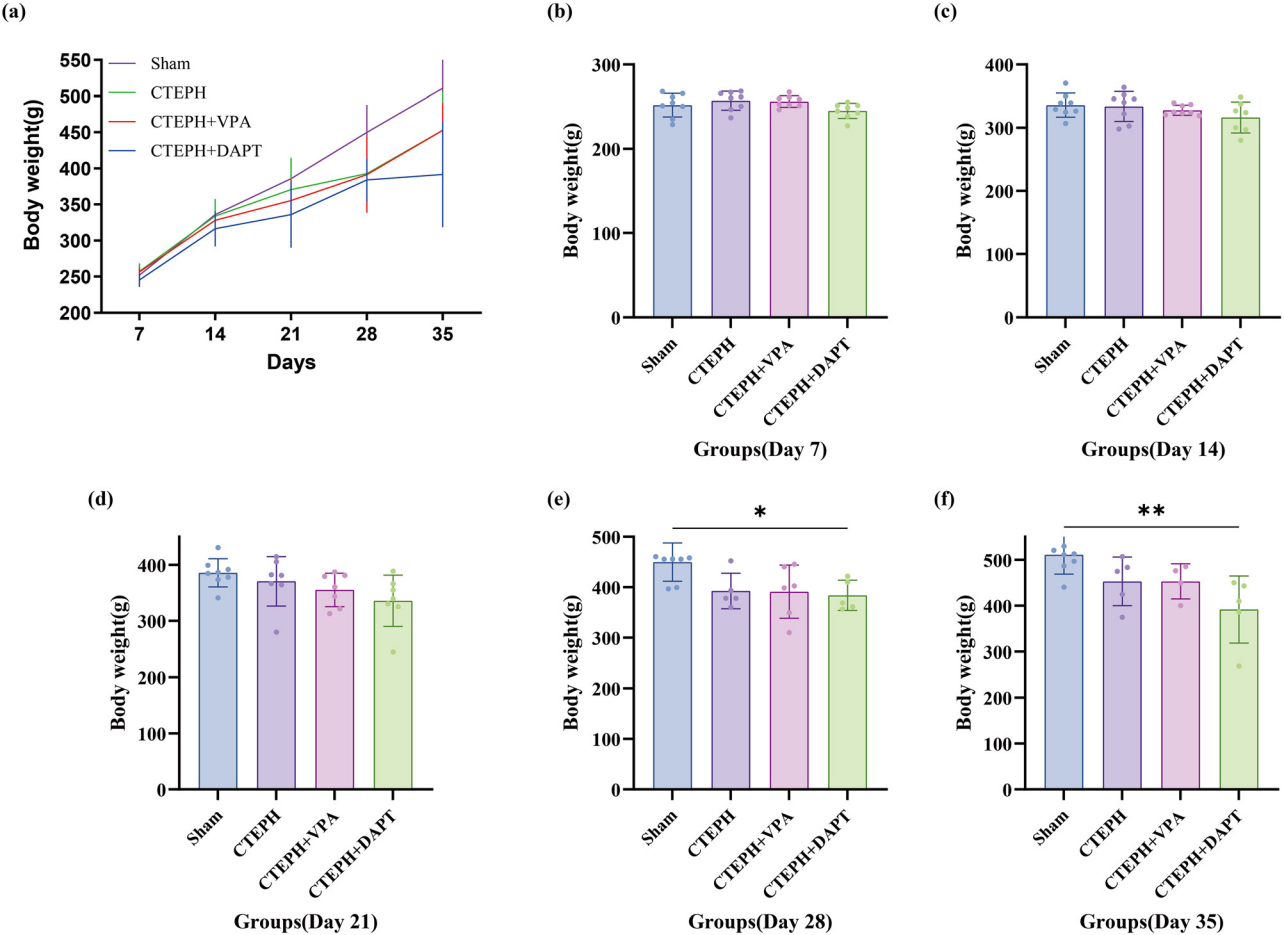
Changes in body weight during the treatment. The body weight of rats was measured on days 7, 14, 21, 28, and 35, and data are shown as mean ± SD (a). And (b), (c), (d), (e), (f) represented the body weight of rats in different groups in each group, and data are shown as mean ± SD In the inter-group difference analysis, the normality of the data for each group was first assessed using the Shapiro-Wilk test. If the data followed a normal distribution, pairwise ANOVA was performed to evaluate the significance of inter-group differences. If the data did not follow a normal distribution, the Kruskal-Wallis test was applied. A *p*-value greater than 0.05 was considered not significant, while a *p*-value less than 0.01 was deemed significant and marked with “**”. For *p*-values between 0.01 and 0.05, significance was indicated with “*”, and these values were annotated in the figures.

The CTEPH + VPA group demonstrated improved overall health compared to the CTEPH model group, characterized by a gradual increase in body weight, although four rats died. Body weight increased by an average of 52.2 % in Sham versus 35.7 % in CTEPH rats by week 4. Rats in the CTEPH + DATP group had a deteriorating condition characterized by dull fur, progressive weight loss, decreased activity, and the death of three rats (Figure 2 and Table S2).

### 3.2 Hemodynamic changes in rats

Compared to the Sham group, rats in the CTEPH model group exhibited a significant increase in mean pulmonary arterial pressure (MPAP) and right ventricular systolic pressure (RVSP), with both comparisons being significant (*p* < 0.05). Compared to the CTEPH model group, rats in the CTEPH + VPA model group also showed a significant increase in MPAP and RVSP (*p* < 0.05). However, compared to the CTEPH + VPA model group, rats in the CTEPH + DAPT model group had a significant decrease in MPAP and RVSP (*p* < 0.05) (Table 1, Table S1, Figure S1, and Figure S2).

**Table 1:** Changes in MPAP (mmHg) and RVSP (mmHg) among different groups.

Groups	MPAP (mmHg)	RVSP (mmHg)
Sham (*n* = 8)	9.79 ± 0.45	13.99 ± 1.02
CTEPH (*n* = 5)	16.66 ± 1.64^a^	19.92 ± 1.99^a^
CTEPH + VPA (*n* = 4)	19.25 ± 1.66^a,b^	26.09 ± 2.34^a,b^
CTEPH + DAPT (*n* = 5)	14.96 ± 1.00^a,b,c^	17.63 ± 2.01^a,c^

^a^Denotes a significant difference compared to the Sham group (*p* < 0.05); ^b^indicates a significant difference compared to the CTEPH model group (*p* < 0.05); ^c^represents a significant difference compared to the CTEPH + VPA group (*p* < 0.05).

### 3.3 HE staining of pulmonary small arteries in rats

**Figure 3:**
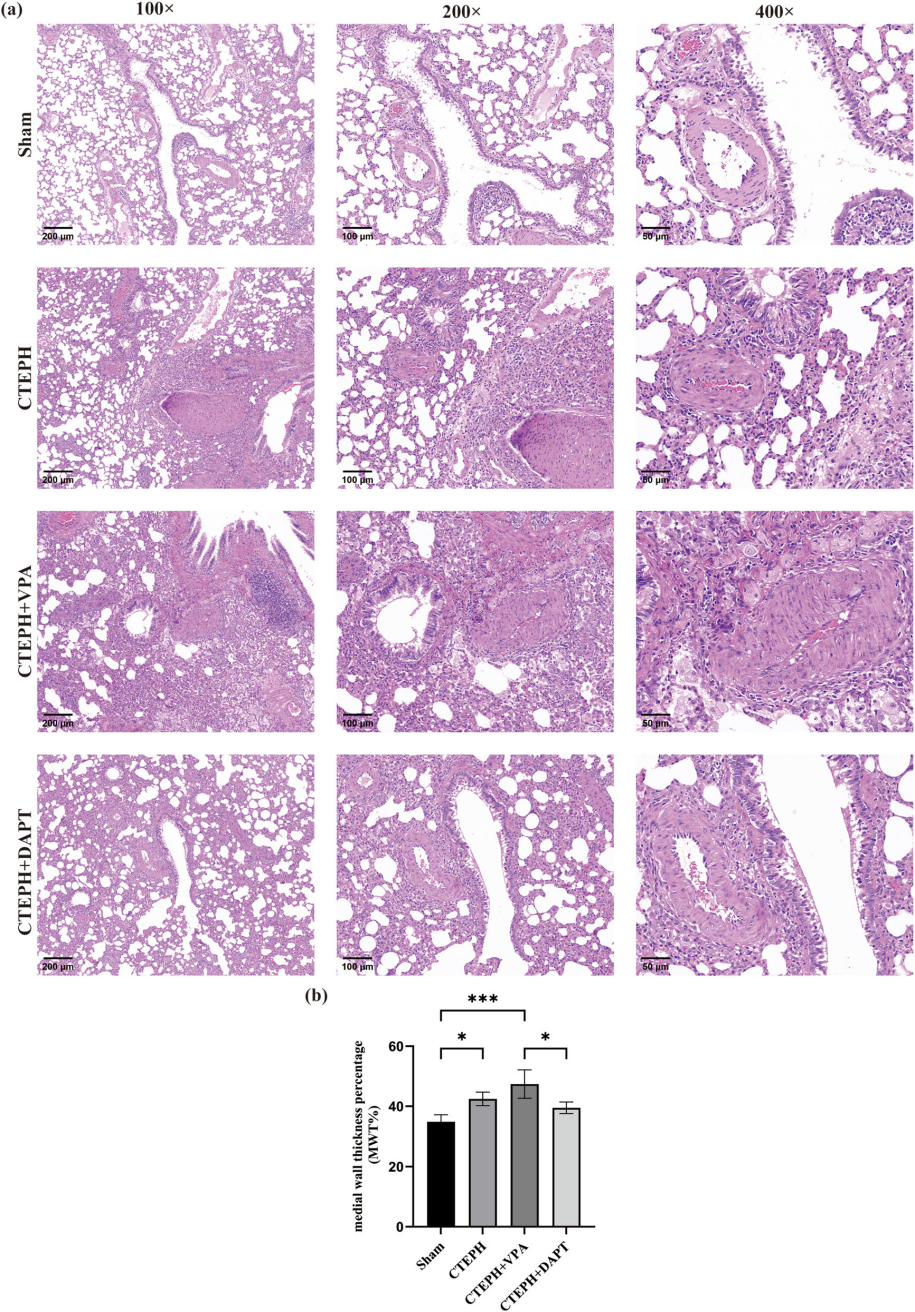
Histological HE staining of lung tissues. (a) HE staining. (b) Comparison of MWT% in small pulmonary arteries among different groups. Data are shown as mean ± SD. *p < 0.05, **p < 0.01, ***p < 0.001, ****p < 0.0001. As shown in the figure, rats in the Sham group had normal lung tissue structure with evenly arranged alveoli, while the CTEPH group exhibited thickened alveolar walls and increased inflammatory cell infiltration around the bronchioles; the CTEPH + VPA group also showed endothelial cell proliferation and thickened arterial walls in the small pulmonary arteries, and the CTEPH + DAPT group had a reduced degree of arterial wall thickening compared to the CTEPH model group.

HE staining results showed that rats in the Sham group had normal lung tissue structure, evenly arranged alveoli, minimal inflammatory cell infiltration around the bronchioles, and no significant changes in the pulmonary small artery endothelium. In contrast, the CTEPH model group exhibited thickened alveolar walls, increased inflammatory cell infiltration around the bronchioles, endothelial cell proliferation in the pulmonary small arteries, and thickened arterial walls. Compared to the CTEPH model group, the CTEPH + VPA group also showed endothelial cell proliferation and thickened arterial walls in the small pulmonary arteries. The CTEPH + DAPT group had a reduced degree of arterial wall thickening compared to the CTEPH model group (Figure 3).

Compared to the Sham group, the MWT% in both the CTEPH model group and the CTEPH + VPA group was significantly increased (*p* < 0.05). Compared to the CTEPH model group, the CTEPH + VPA group also showed a significant increase in MWT% (*p* < 0.05). Furthermore, compared to the CTEPH + VPA group, the CTEPH + DAPT group had a significant decrease in MWT% (*p* < 0.05) (Table 2, Table S3, and Figure 3(b)).

**Table 2:** Comparison of medial wall thickness percentage (MWT%) in small pulmonary arteries among different groups.

Groups	MWT%
Sham	34.93 % ± 2.23
CTEPH	42.47 % ± 2.24^a^
CTEPH + VPA	47.39 % ± 4.73^a,b^
CTEPH + DAPT	39.50 % ± 1.91^c^

The percentage of MWT relative to ED (MWT%) was calculated using the formula: MWT% = (2 × MWT/ED) × 100 %. ^a^Denotes a significant difference compared to the Sham group (*p* < 0.05); ^b^indicates a significant difference compared to the CTEPH model group (*p* < 0.05); ^c^represents a significant difference compared to the CTEPH + VPA group (*p* < 0.05).

### 3.4 Immunohistochemical results

#### 3.4.1 PCNA protein

**Figure 4:**
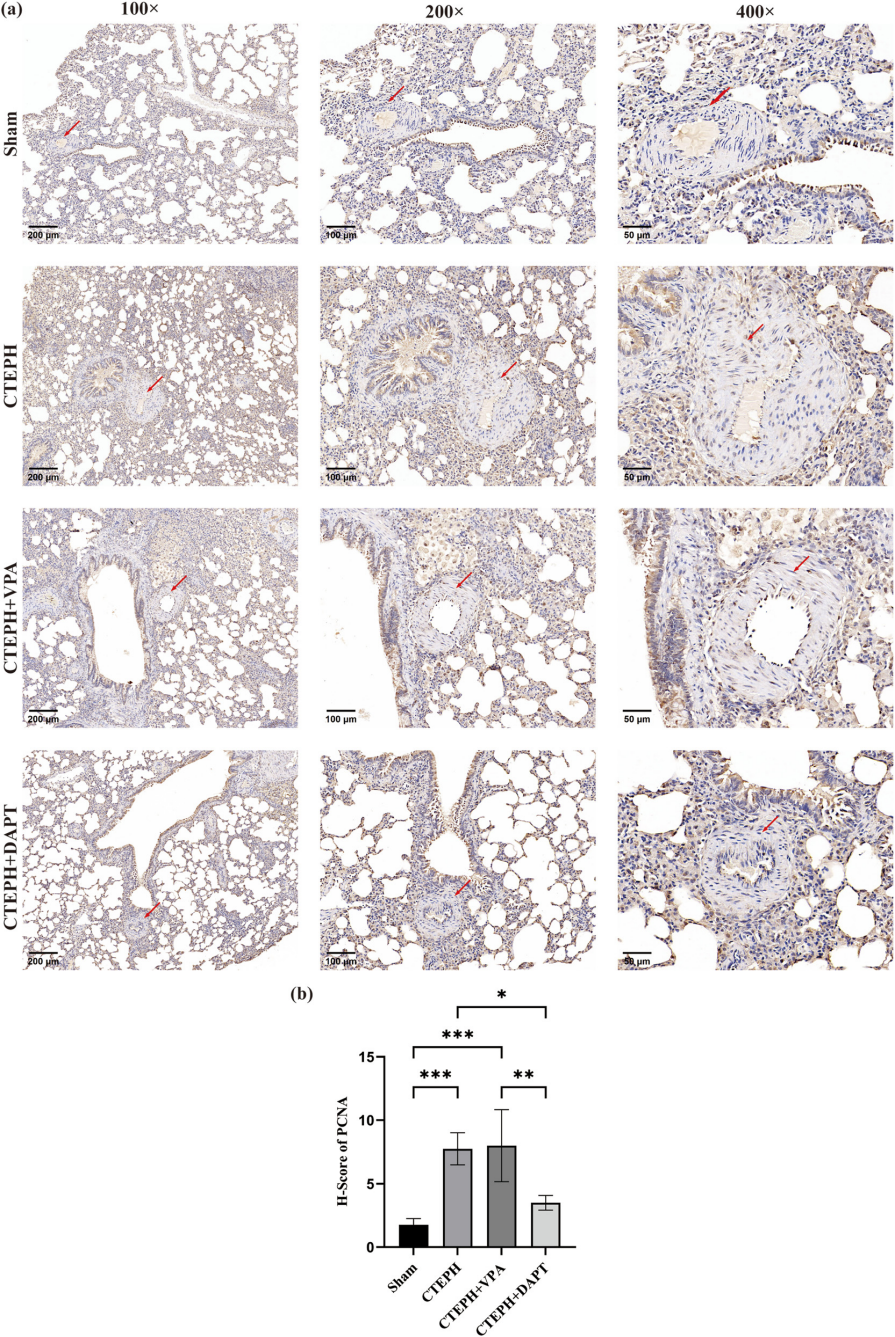
Immunohistochemical staining of PCNA in rat lung tissues (a) immunohistochemical staining of PCNA. (b) H-Score of PCNA. Data are shown as mean ± SD. *p < 0.05, **p < 0.01, ***p < 0.001, ****p < 0.0001. As shown in the figure, a relatively lower expression of PCNA was observed. The CTEPH model group showed a higher intensity of protein expression. In comparison to the CTEPH model group, the CTEPH + VPA group exhibited a slight increase in the number of positive cells and a modest rise in protein expression intensity. In the CTEPH + DAPT group, we observed a lower protein expression.

PCNA protein is expressed in the vascular walls of lung tissue, with brown-yellow granules indicating positive expression, which can be observed in the cell nuclei and cytoplasm. In the Sham group, there were relatively few positive cells within the blood vessels of the lung tissue, and a lower expression of PCNA was observed. Compared to the Sham group, the CTEPH model group showed an increased number of positive cells and a higher intensity of protein expression. In comparison to the CTEPH model group, the CTEPH + VPA group exhibited a slight increase in the number of positive cells and a modest rise in protein expression intensity. Conversely, the CTEPH + DAPT group had fewer positive cells and lower protein expression intensity than the CTEPH model group (Figure 4).

#### 3.4.2 *α*-SMA protein

**Figure 5:**
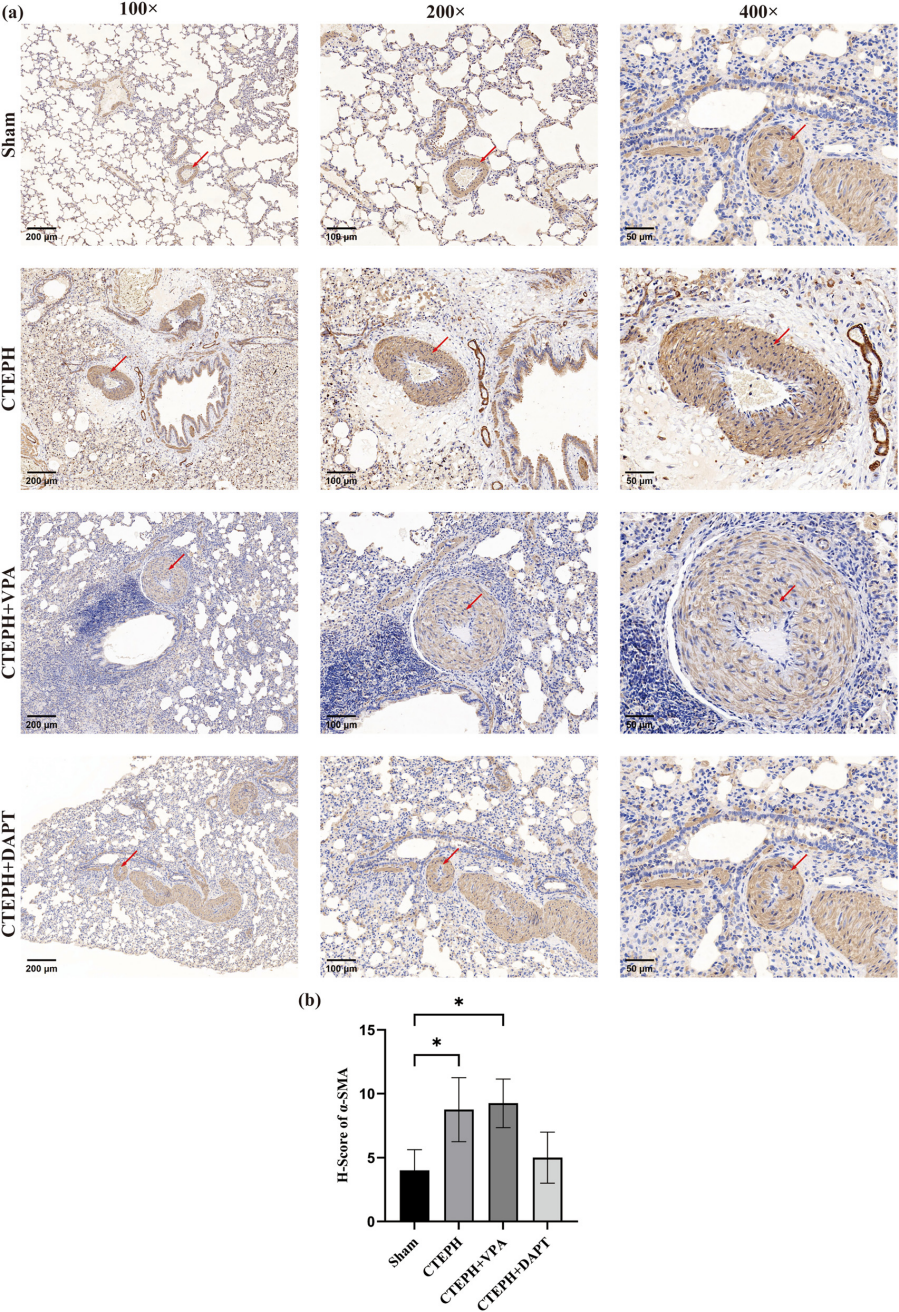
Immunohistochemical staining of *α*-SMA. (a) Immunohistochemical staining of *α*-SMA. (b) H-Score of *α*-SMA. Data are shown as mean ± SD. *p < 0.05, **p < 0.01, ***p < 0.001, ****p < 0.0001. As shown in the figure, compared to the Sham group, the CTEPH model group exhibited thickening of the pulmonary small artery walls. Compared to the CTEPH model group, the CTEPH + VPA group showed a slight thickening of the pulmonary small artery walls, a marginal increase in expression area, and a modest rise in protein expression intensity.

*α*-SMA protein is localized in the cytoplasm of vascular smooth muscle cells, with brown-yellow granules indicating positive expression. In the Sham group, *α*-SMA protein expression was observed in the pulmonary small arteries, with a limited expression area and low intensity. Compared to the Sham group, the CTEPH model group exhibited thickening of the pulmonary small artery walls, with an increased expression area and higher protein expression intensity. Compared to the CTEPH model group, the CTEPH + VPA group showed a slight thickening of the pulmonary small artery walls, a marginal increase in expression area, and a modest rise in protein expression intensity. Conversely, the CTEPH + DAPT group had reduced wall thickness, a decreased expression area, and lower protein expression intensity than the CTEPH model group (Figure 5).

#### 3.4.3 Comparison of PCNA and *α*-SMA protein expression scores

Compared to the Sham group, the PCNA and *α*-SMA scores were significantly elevated in the CTEPH model and CTEPH + VPA groups (*p* < 0.050), and scores were calculated as described in Section 2.6. In contrast, the PCNA and *α*-SMA scores in the CTEPH + DAPT group were significantly reduced compared to the CTEPH model group (*p* < 0.05). Additionally, the PCNA and *α*-SMA scores in the CTEPH + DAPT group were significantly lower than those in the CTEPH + VPA group (*p* < 0.05) (Table 3 and Table S4, Figures 4(b) and 5(b)).

**Table 3:** Comparison of PCNA and *α*-SMA protein expression scores in small pulmonary arteries among different groups.

Groups	PCNA	*α*-SMA
Sham	1.75 ± 0.50	4.00 ± 1.63
CTEPH	7.75 ± 1.26^a^	8.75 ± 2.50^a^
CTEPH + VPA	8.00 ± 2.83^a^	9.25 ± 1.89^a^
CTEPH + DAPT	3.50 ± 0.58^b,c^	5.00 ± 2.00

^a^Denotes a significant difference compared to the Sham group (*p* < 0.05); ^b^indicates a significant difference compared to the CTEPH model group (*p* < 0.05); ^c^represents a significant difference compared to the CTEPH + VPA group (*p* < 0.05).

### 3.5 Expression of Notch1, Notch3, Jagged1, and Hes1 proteins in pulmonary small arteries of each group

**Figure 6:**
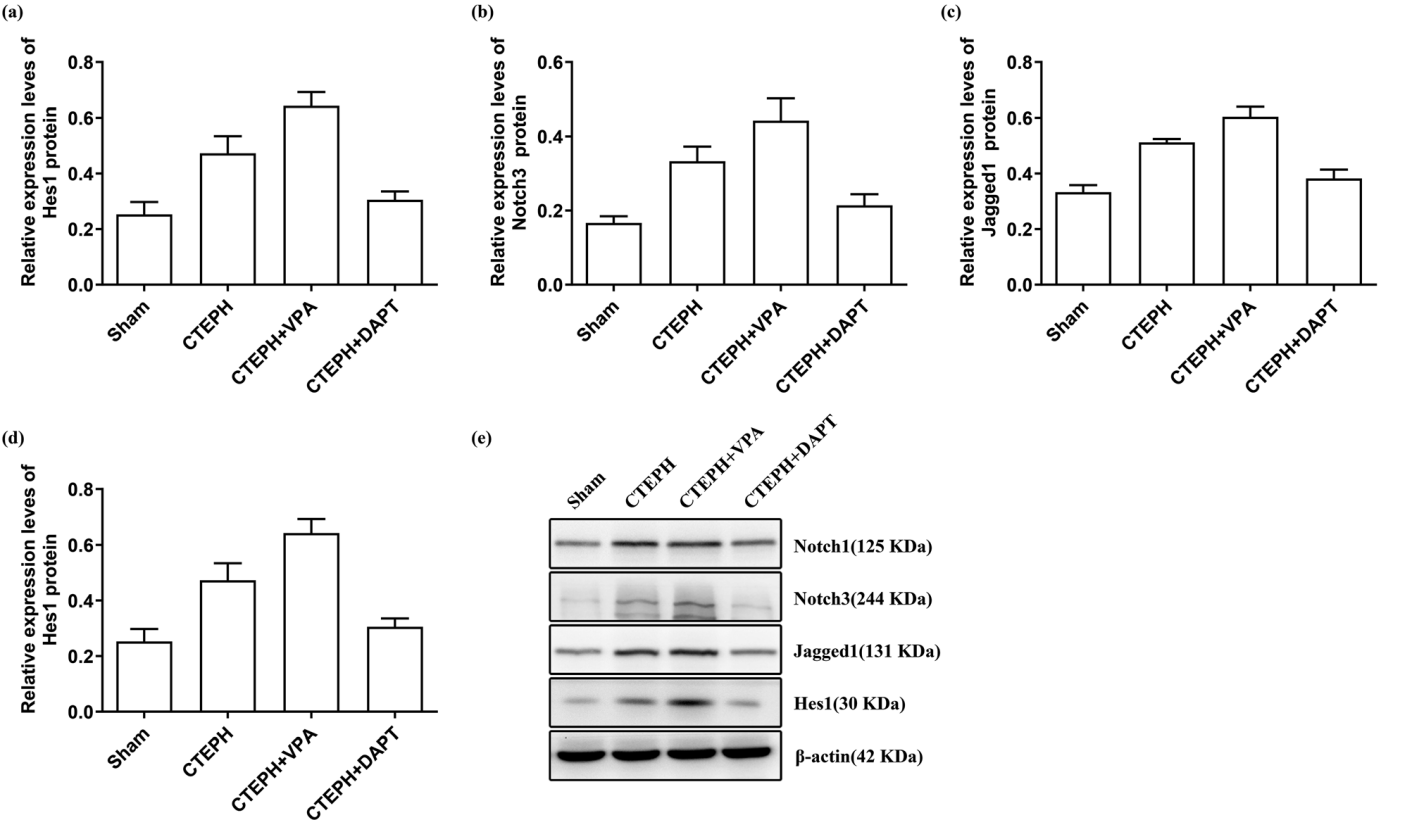
Expression of Notch1 (a), Notch3 (b), Jagged1 (c), and Hes1 (d) in different groups. Data were collected from triplicate experiments and are shown as the means ± SD (e) western blot analysis of Notch1, Notch3, Jagged1, and Hes1. Loading control was assessed by *β*-actin. The position of molecular weight markers is shown in KDa.

Compared to the Sham group, the levels of Notch1, Notch3, Jagged1, and Hes1 proteins were significantly elevated in both the CTEPH model and CTEPH + VPA groups (*p* < 0.05). Compared to the CTEPH model group, the CTEPH + VPA group also showed significantly increased levels of Notch1, Notch3, Jagged1, and Hes1 proteins (*p* < 0.05). In contrast, the CTEPH + DAPT group exhibited significantly reduced levels of Notch1, Notch3, Jagged1, and Hes1 proteins (*p* < 0.05). Compared to the CTEPH + VPA group, the CTEPH + DAPT group had significantly lower levels of these proteins (*p* < 0.05) (Table 4 and Figure 6).

**Table 4:** Protein expression levels in small pulmonary arteries among different groups.

Groups	Notch1	Notch3	Jagged1	Hes1
Sham	0.435 ± 0.033	0.167 ± 0.018	0.333 ± 0.025	0.253 ± 0.044
CTEPH	0.625 ± 0.042^a^	0.334 ± 0.039^a^	0.512 ± 0.012^a^	0.473 ± 0.061^a^
CTEPH + VPA	0.717 ± 0.049^a,b^	0.443 ± 0.060^a,b^	0.604 ± 0.035^a,b^	0.643 ± 0.049^a,b^
CTEPH + DAPT	0.493 ± 0.060^b,c^	0.215 ± 0.030^b,c^	0.383 ± 0.032^b,c^	0.306 ± 0.030^b,c^

^a^Denotes a significant difference compared to the Sham group (*p* < 0.05); ^b^indicates a significant difference compared to the CTEPH model group (*p* < 0.05); ^c^represents a significant difference compared to the CTEPH + VPA group (*p* < 0.05).

## 4 Discussion and conclusion

Chronic thromboembolic pulmonary hypertension poses a significant disease burden, and despite the symptomatic relief provided by surgical, interventional, and pharmacological treatments, the prognosis remains poor. Once pulmonary vascular remodeling occurs, it becomes nearly irreversible, making it a critical and challenging aspect of CTEPH management [32]. Therefore, investigating the mechanisms underlying pulmonary vascular remodeling is paramount for advancing CTEPH therapy.

Reliable animal models are essential for deepening our understanding of CTEPH and elucidating its pathogenesis. In this study, a rat model of CTEPH was established using polystyrene microspheres, resulting in a mortality rate of 37.5–50 %, consistent with previously reported literature [33]. The primary causes of death were ischemic stroke or acute heart failure induced by embolic occlusion. Pulmonary artery pressure (PAP) and right ventricular systolic pressure (RVSP) serve as objective indicators for assessing the success of pulmonary hypertension (PH) models [34]. This study demonstrated significantly elevated pulmonary artery and right ventricular systolic pressures, confirming the successful establishment of a CTEPH model in rats. The percentage of medial thickness (MT%) in small pulmonary arteries is a common indicator of pulmonary vascular remodeling [35]. PCNA, a stable cell cycle-related nuclear protein, reflects cell proliferation. *α*-SMA, a molecular marker of smooth muscle phenotype expressed by pulmonary smooth muscle cells, indicates thickening of the medial layer and muscularization of pulmonary arteries [36].

In this study, we found that compared to the Sham group, HE staining revealed a significant increase in wall thickness and WT% in the CTEPH model group. Increased numbers of PCNA-positive cells, higher expression of *α*-SMA protein, and elevated immunohistochemical scores for PCNA and *α*-SMA were observed in the CTEPH model group. This suggests excessive proliferation of smooth muscle cells in CTEPH rats, leading to thickening of the pulmonary arteriole walls and pulmonary vascular remodeling.

In normal adult pulmonary vasculature, PASMCs usually maintain a differentiated state, performing contractile functions without migration. However, during development, tissue injury, and vascular remodeling, PASMCs adjust their phenotype in response to tissue demands, exiting their quiescent state [37]. Research has indicated that PASMCs in CTEPH patients exhibit tumor-like proliferation and apoptosis resistance, potentially contributing to pulmonary vascular remodeling [38]. Notch signaling may promote PASMC proliferation via Hes-mediated transcriptional regulation of cell cycle genes [39]. In addition, studies on hypoxic CTEPH rats have shown increased numbers of PASMCs in the mitotic phase, excessive smooth muscle cell proliferation, and disrupted balance between proliferation and apoptosis, leading to vascular remodeling and pulmonary hypertension [12], 40]. We postulate that PASMCs in CTEPH rats exhibit tumor-like characteristics, uncontrolled replication potential, evasion of growth inhibitors, DNA instability, activation of specific signaling pathways, and altered cellular metabolism, resulting in increased volume, number, and migration of PASMCs, leading to muscularization, narrowing, and increased vascular tension of the small pulmonary arteries. Additionally, extracellular matrix protein deposition causes vascular wall thickening, increasing resistance in the small pulmonary arteries and ultimately leading to vascular remodeling. Notch signaling may promote PASMC proliferation via Hes-mediated transcriptional regulation of cell cycle genes [39]. Over the past two decades, researchers worldwide have explored the various biological roles of the Notch signaling pathway in respiratory system development, homeostasis, and regeneration, providing valuable insights into the pathogenesis of challenging respiratory diseases [41], 42]. Disruptions in Notch signaling can lead to cancerous [43]. Jagged-1, one of the ligands for the Notch receptor, triggers the Notch signaling pathway through its extracellular binding domain [44]. Currently, Hes1, Hes5, and Hes7 are considered Notch effectors [45]. Recent findings in pulmonary arterial hypertension (PAH) have highlighted the role of the Notch pathway [46], 47].

Notch3 expression in the lungs is restricted to smooth muscle cells and strongly correlates with the severity of PAH in both human subjects and mouse models [48]. Lung tissues from PAH patients exhibit higher Notch3 expression than controls [49], suggesting a significant correlation with Notch3 protein concentration. To investigate the involvement of the Notch signaling pathway in CTEPH-induced pulmonary vascular remodeling, this study further employed the Notch pathway activator VPA and inhibitor DAPT in experiments with CTEPH rats.

HE staining in this study showed that compared to the CTEPH model group, the CTEPH + VPA group exhibited increased endothelial cell proliferation and wall thickening in small pulmonary arteries, with a significant rise in MWT%. In contrast, the CTEPH + DAPT group showed no significant decrease in MWT%. Immunohistochemical analysis revealed that, compared to the CTEPH model group, the CTEPH + DAPT group had significantly lower PCNA and *α*-SMA scores. These results may be attributed to VPA being a non-specific Notch pathway activator that might affect endothelial cells in small pulmonary arteries [50]. In contrast, DAPT acts on PASMCs, inhibiting Notch protein cleavage, NICD nuclear translocation, and NICD signaling, thereby exerting strong inhibitory effects on Notch signaling.

Western blot analysis demonstrated that in CTEPH rats injected with VPA, levels of Notch1, Notch3, Jagged1, and Hes1 proteins were significantly higher compared to the CTEPH group. In contrast, these protein levels were markedly lower in CTEPH rats injected with DAPT. This indicates that elevated Notch1, Notch3, Jagged1, and Hes1 proteins and increased Notch/Jagged/Hes signaling pathway activity promote PASMC proliferation and contribute to pulmonary vascular remodeling in CTEPH. Conversely, DAPT application can inhibit Notch signaling, reducing vascular remodeling [51]. The mechanisms through which the Notch signaling pathway affects pulmonary vascular remodeling (especially in smooth muscle cells) may include: (1) promoting smooth muscle cells to enter the S phase, losing growth inhibition and cell cycle arrest, leading to excessive proliferation [52]; (2) inhibiting smooth muscle cell apoptosis, extending their survival time; (3) promoting the transformation of endothelial cells and fibroblasts into smooth muscle cells; (4) inhibiting the transcriptional regulation of various smooth muscle cell marker genes and their expression, thereby inhibiting differentiation and promoting conversion to a synthetic phenotype [53].

Recent findings have shown that Notch can reverse pulmonary vascular remodeling in PAH animal models without local or systemic toxicity [53], 54]. Further exploration of the Notch signaling pathway’s influence on various phenotypes and its relationship with CTEPH pathogenesis is needed to uncover potential therapeutic targets. Hence, targeting the Notch signaling pathway with *γ*-secretase inhibitors, such as DAPT, could be explored in clinical trials for CTEPH. Given that our study demonstrated the activation of Notch signaling promotes pulmonary artery smooth muscle cell proliferation and contributes to pulmonary vascular remodeling in a rat model of CTEPH, inhibiting this pathway with DAPT mitigated these effects. This suggests that similar therapeutic strategies might have the potential to alleviate vascular remodeling in human CTEPH patients. In addition, our findings demonstrate that Notch signaling drives PASMC proliferation and vascular remodeling in CTEPH, and its inhibition with DAPT offers a promising therapeutic strategy.

However, our study has some limitations. Firstly, the rat model used in this study cannot fully replicate human physiological characteristics due to the distant evolutionary relationship [55]. Although our study found that the levels of Notch1, Notch3, Jagged1, and Hes1 proteins were elevated in CTEPH rats, and that the activation of the Notch signaling pathway promotes the proliferation of PASMCs, contributing to PVR in CTEPH, these findings may not be directly applicable to humans. The differences in species could lead to variations in the response of PASMCs to Notch signaling activation and the effectiveness of DAPT treatment [56].

Moreover, the rat model may not fully capture the complexity of human CTEPH, which is influenced by a variety of genetic and environmental factors [57]. Future studies should consider using more human-relevant models, such as human tissue samples or humanized animal models, to validate our findings and further explore the role of the Notch signaling pathway in CTEPH.

## Supplementary Material

Supplementary Material

Supplementary Material

Supplementary Material

Supplementary Material

Supplementary Material

Supplementary Material
