# Supplementary figures and images for: Notch signaling enhances PASMC proliferation and vascular remodeling in CTEPH
Source: Open Life Sci. 2026 Feb 25;21(1):20251251. doi: 10.1515/biol-2025-1251 (PMC12949630; doi:10.1515/biol-2025-1251)

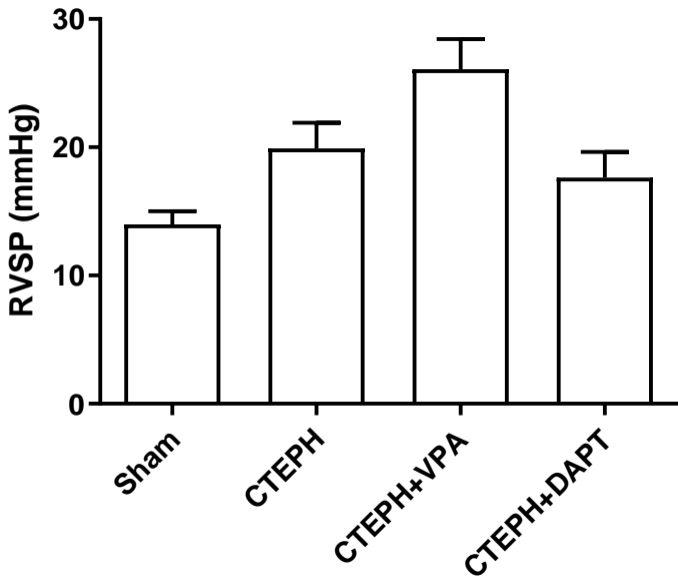

Supplement: Supplementary file 1 — Supplementary Material [file j_biol-2025-1251_suppl_001.pdf]

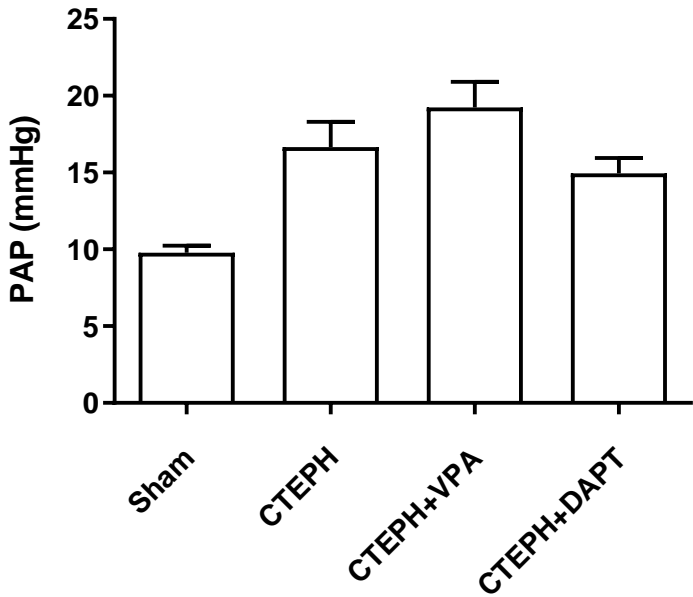

Supplement: Supplementary file 5 — Supplementary Material [file j_biol-2025-1251_suppl_005.pdf]
